# Stroke triggers nigrostriatal plasticity and increases alcohol consumption in rats

**DOI:** 10.1038/s41598-017-02714-z

**Published:** 2017-05-31

**Authors:** Cathy C. Y. Huang, Tengfei Ma, Emily A. Roltsch Hellard, Xuehua Wang, Amutha Selvamani, Jiayi Lu, Farida Sohrabji, Jun Wang

**Affiliations:** Department of Neuroscience and Experimental Therapeutics, College of Medicine, Texas A&M University Health Science Center, Bryan, TX 77807 USA

## Abstract

Excessive alcohol consumption is a known risk factor for stroke, but the effect of stroke on alcohol intake is unknown. The dorsomedial striatum (DMS) and midbrain areas of the nigrostriatal circuit are critically associated to stroke and alcohol addiction. Here we sought to explore the influence of stroke on alcohol consumption and to uncover the underlying nigrostriatal mechanism. Rats were trained to consume alcohol using a two-bottle choice or operant self-administration procedure. Retrograde beads were infused into the DMS or midbrain to label specific neuronal types, and ischemic stroke was induced in the dorsolateral striatum (DLS). Slice electrophysiology was employed to measure excitability and synaptic transmission in DMS and midbrain neurons. We found that ischemic stroke-induced DLS infarction produced significant increases in alcohol preference, operant self-administration, and relapse. These increases were accompanied by enhanced excitability of DMS and midbrain neurons. In addition, glutamatergic inputs onto DMS D1-neurons was potentiated, whereas GABAergic inputs onto DMS-projecting midbrain dopaminergic neurons was suppressed. Importantly, systemic inhibition of dopamine D1 receptors attenuated the stroke-induced increase in operant alcohol self-administration. Our results suggest that the stroke-induced DLS infarction evoked abnormal plasticity in nigrostriatal dopaminergic neurons and DMS D1-neurons, contributing to increased post-stroke alcohol-seeking and relapse.

## Introduction

Stroke is a major cause of death and adult disability^1^. In ischemic stroke, a lack of blood flow to the brain results in the damage of neurons and their connected neural circuits, leading to behavioral impairments^2^. After a stroke, the brain shows unusual neuroplasticity that can accelerate recovery, or cause unexpected behavioral changes^2–4^. Little is known about how ischemic stroke alters addictive behaviors and their underlying mechanisms. Given that drug and alcohol addiction are well-known and prevalent risk factors for stroke^5, 6^, studying stroke-induced changes in addictive behaviors could help to prevent stroke recurrence.

Clinical analysis reveals that the majority of strokes damage subcortical regions such as the basal ganglia^7^, which is known to play vital roles in drug and alcohol addiction and is regulated by dopamine signals^8–10^. Dopaminergic neurons in the substantia nigra pars compacta (SNc) that have been implicated in alcohol addiction^11^, innervate the dorsal striatum, the largest component of the basal ganglia^12^. The dorsal striatum is involved in drug and alcohol addiction^9, 13–16^ and it can be divided into the dorsomedial striatum (DMS) and the dorsolateral striatum (DLS). Striatal neurons expressing dopamine D1 receptors (D1-neurons) positively control drug and alcohol addiction in a glutamatergic- and dopaminergic-dependent manner^14, 15, 17^. However, it is unclear whether or how the effects of ischemic stroke on alcohol addiction involve changes in the activity of this nigrostriatal pathway.

In this study, we combined behavioral and electrophysiological strategies to investigate how stroke-induced infarction of the DLS changes neural activity in the nigrostriatal pathway and alcohol consumption in rats. Behaviorally, we observed that ischemic stroke increased the preference for alcohol, alcohol seeking, and relapse. Electrophysiologically, we observed that the excitabilities of both DMS and midbrain neurons were augmented post-stroke. Importantly, the glutamatergic strength onto DMS D1-neurons was enhanced after ischemia, whereas GABAergic activity in DMS-projecting dopamine neurons was attenuated. Finally, pharmacological inhibition of D1 receptors reduced post-stroke alcohol consumption. Our findings provide insights into the mechanisms underlying the impact of stroke on neuroplasticity in the basal ganglia and on alcohol intake. These mechanistic insights may provide novel treatment targets to promote recovery and prevent stroke recurrence.

## Results

### Stroke increases home-cage alcohol preference in rats

**Figure 1:**
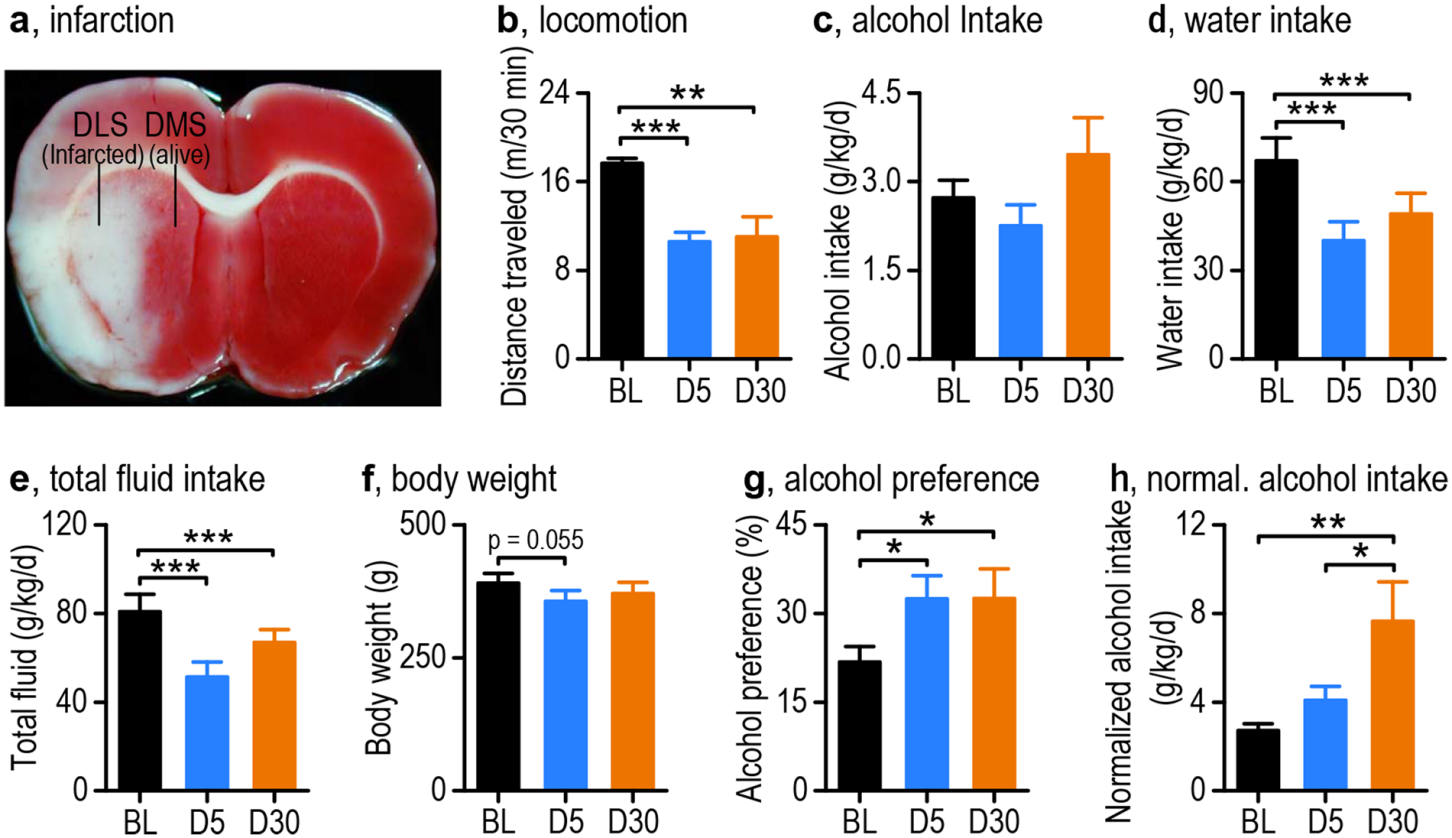
Ischemic stroke induces an increase in home-cage alcohol preference in rats. Sprague-Dawley rats were trained to consume 20% alcohol until a stable baseline was achieved prior to induction of ischemic stroke. (**a**) Sample image of 2,3,5-triphenyltetrazolium chloride (TTC)-stained tissue, showing that ischemic stroke was induced in the DLS but not in the DMS. (**b**) Ischemic stroke resulted in a decrease in locomotion on day 5 (D5) and day 30 (D30) post-stroke, as compared to baseline (BL). **p < 0.01, ***p < 0.001. n = 19 (BL), 19 (D5), and 7 (D30) rats. (**c**) Voluntary alcohol intake is shown before (BL), on D5, and on D30 post-stroke. n = 19 (BL), 19 (D5), and 14 (D30) rats. (**d**,**e**) Ischemic stroke led to significant decreases in water intake (**d**) and total fluid intake (**e**) on both D5 and D30. ***p < 0.001. n = 19 (BL), 19 (D5), and 14 (D30) rats. (**f**) Ischemic stroke caused a marginal decrease in body weight on D5 but not on D30. n = 19 (BL), 19 (D5), and 14 (D30) rats. (**g**) Ischemic stroke increased alcohol preference on D5 and D30 post-stroke, as compared to BL. *p < 0.05. n = 19 (BL), 19 (D5), and 13 (D30) rats. (**h**) Ischemic stroke caused an increased in alcohol intake (normalized to locomotor activity) on D30, as compared to BL and D5. *p < 0.05, **p < 0.01. n = 19 (BL), 19 (D5), and 14 (D30) rats. (**b–h**) Statistics were conducted using One-Way RM ANOVA followed by SNK test.

To examine whether ischemic stroke altered alcohol consumption, we first trained Sprague-Dawley (SD) rats for 8 weeks to consume 20% alcohol using the intermittent-access 2-bottle choice procedure^18, 19^. Endothelin-1 was then infused into the left hemisphere of the brain, close to the middle cerebral artery, in order to induce ischemic stroke^20, 21^. We selected the endothelin-1 model, rather than other models, because it is less invasive and causes a reliable and focal infarct in the lateral striatum^20, 22^ (Fig. 1a). Alcohol consumption and locomotion were measured before and after stroke induction. As expected, there was a significant effect of stroke on open field locomotion during this period (Fig. 1b; *F*_(2,24)_ = 22.79, *p* < 0.0001). We found a significant decrease in locomotion on day 5 (*q* = 9.40, *p* = 0.00013) and day 30 (*q* = 4.70, *p* = 0.0030), as compared to the pre-stroke baseline.

Although we only found a trend toward increased alcohol intake at day 30 post-stroke (Fig. 1c; 2.73 ± 0.30 g/kg/24 h pre-stroke and 3.46 ± 0.62 g/kg/24 h post-stroke; *F*_(2,31)_ = 2.42, *p* = 0.11), we did find a significant effect of stroke on water intake (Fig. 1d; *F*_(2,231)_ = 23.83, *p* = 0.000) and on total fluid intake (Fig. 1e; *F*_(2,31)_ = 22.19, *p* = 0.000). Both the water intake and total fluid intake were significantly decreased on D5 and D30, as compared to baseline (water/D5: *q* = 9.02, *p* = 0.00013; water/D30: *q* = 7.35, *p* = 0.00015; total fluid/D5: *q* = 9.22, *p* = 0.000; total fluid/D30: *q* = 5.83, *p* = 0.0004). These changes were accompanied with a marginal reduction of body weight on D5 but not on D30 (Fig. 1f; *q* = 3.42, *p* = 0.055 and *q* = 0.88, *p* = 0.54, respectively). Importantly, the decreased water intake led to a significant change in alcohol preference (Fig. 1g; *F*_(2,30)_ = 5.38, *p* = 0.010). We found a significant increase in alcohol preference that started on day 5 and persisted to day 30, as compared to baseline (*q* = 3.71, *p* = 0.014 and *q* = 4.11, *p* = 0.018, respectively). While alcohol preference was unlikely to be influenced by stroke-induced impairment of locomotion, this may affect voluntary alcohol intake. We therefore normalized this parameter to locomotion levels, revealing a significant effect of stroke on alcohol consumption (Fig. 1h; *F*_(2,31)_ = 5.79, *p* = 0.0073). We then found a significant increase in alcohol consumption on day 30, as compared to baseline and day 5 (*q* = 4.77, *p* = 0.0057 and *q* = 3.41, *p* = 0.022, respectively). These alterations seemed unlikely induced by stereotaxic infusion surgeries since infusion of retrograde beads^23–25^ did not significantly alter locomotion on D15 (Supplementary Fig. S1a; *t*_(14)_ = 1.91, *p* = 0.076) or alcohol intake from D7 onwards (Supplementary Fig. S1b; *F*_(4, 87)_ = 2.15, *p* = 0.082). These results suggest that while the animals that underwent stroke decreased their total fluid intake by reducing water consumption, they maintained their alcohol intake level leading to increased preference for alcohol over water. Collectively, these results indicate that stroke induces an immediate (5 days) and long-term (30 days) increase in alcohol preference.

### Stroke increases alcohol-seeking behavior in rats

SD rats were trained to self-administer 20% alcohol under the fixed ratio 3 (FR3) schedule in operant chambers^19, 26, 27^. The stroke group of rats was infused with endothelin-1, as described above, and the sham group was infused with saline. The lever presses for alcohol and locomotion were monitored before and after stroke induction.

**Figure 2:**
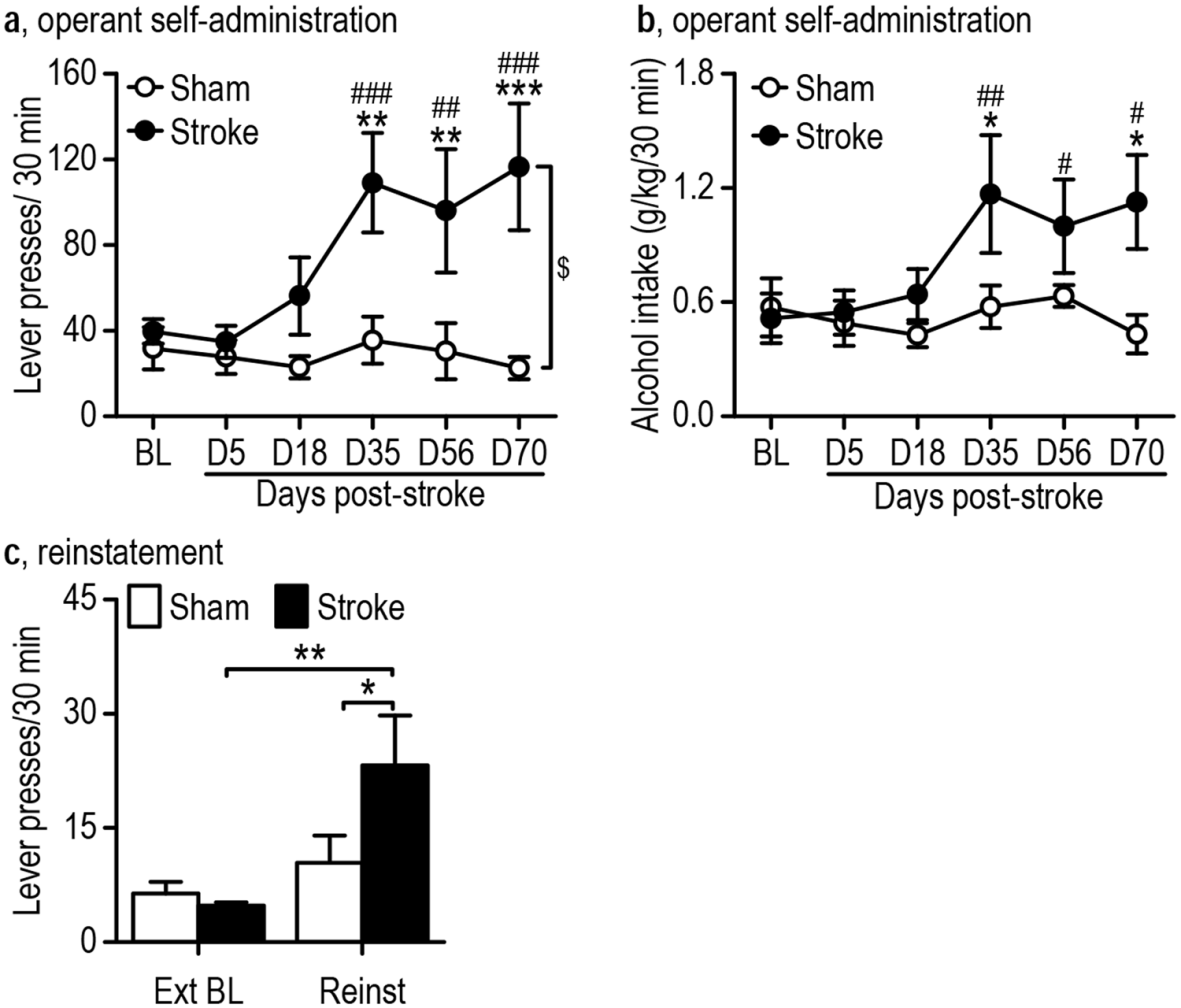
Ischemic stroke enhances operant alcohol self-administration and relapse. Sprague-Dawley rats were trained to consume 20% alcohol in operant chambers until a steady baseline (BL) was established. Stroke or sham control surgery was performed as described in the Experimental Procedures section. (**a**) Ischemic stroke resulted in significantly more lever presses on D35, D56, and D70 post-stroke as compared to sham or BL levels. ^$^ *p* < 0.05, two-way RM ANOVA; **p < 0.01 and ***p < 0.001 compared to sham by post-hoc SNK test; ^##^ *p* < 0.01 and ^###^ *p* < 0.001 compared to stroke BL by post-hoc SNK test. n = 6 (sham) and 5 (stroke) rats. (**b**) Ischemic stroke produced a significant increase in alcohol intake. *p < 0.05 vs. sham, ^#^ *p* < 0.05 vs. stroke BL, and ^##^ *p* < 0.01 vs. stroke BL, two-way RM ANOVA followed by the post-hoc SNK test. n = 6 rats per group. (**c**) Stroke increased alcohol-induced reinstatement of lever presses. *p < 0.05 and **p < 0.01, two-way RM ANOVA followed by the post-hoc SNK test. n = 7 (sham) and 5 (stroke) rats.

Consistent with the 2-bottle choice data, the stroke and sham groups performed similarly on days 5–18 (Fig. 2a); however, we found a significant effect of stroke (*F*_(1,46)_ = 8.65, *p* = 0.015), day (*F*_(5,46)_ = 5.55, *p* = 0.0004) and a stroke x day interaction effect (*F*_(5,74)_ = 5.49, *p* = 0.0005; Two-Way RM ANOVA). Post-hoc analysis revealed an increase in lever presses at post-stroke days 35, 56, and 70, when compared to sham controls (*q* = 4.85, *p* = 0.0019; *q* = 4.28, *p* = 0.0051; and *q* = 6.04, *p* = 0.00030, respectively) and pre-stroke baseline (*q* = 6.05, *p* = 0.00067; *q* = 4.89, *p* = 0.0034; and *q* = 6.57, *p* = 0.00039 respectively). In addition, we also saw a significant increase in alcohol intake (Fig. 2b; day: *F*_(5,46)_ = 3.25, *p* = 0.014; day x stroke interaction: *F*_(5,46)_ = 2.42, *p* = 0.049). Consistent with the lever press data, the stroke and sham groups consumed similarly between days 5 and 18; however, we also observed a significant increase in alcohol intake on post-stroke days 35 and 70, as compared to sham controls (*q* = 3.61, *p* = 0.015 and *q* = 3.54, *p* = 0.017, respectively) and a significant increase in alcohol intake on post-stroke days 35, 56, and 70, when compared to the pre-stroke baseline (*q* = 5.10, *p* = 0.0094; *q* = 3.89, *p* = 0.033; and *q* = 4.38, *p* = 0.017, respectively). Collectively, these results indicate that the motivation of these rats to seek alcohol increased from day 35 post-stroke onwards, and this effect lasted for at least another 35 days.

In rats tested using the operant self-administration procedure, we investigated the effect of stroke on the reinstatement of alcohol seeking (Fig. 2c; day: *F*_(1,10)_ = 13.33, *p* = 0.0045; day × treatment interaction: *F*_(1,10)_ = 5.48, *p* = 0.041). The operant behavior was extinguished over 9 days, followed by an alcohol reinstatement test on day 10^27^. Although we observed only a trend toward an increase in lever presses during reinstatement in the sham animals as compared to the extinction baseline (6.40 ± 1.51 extinction baseline lever presses and 10.43 ± 3.56 reinstatement lever presses; *q* = 1.44, *p* = 0.33), we did observe a significant increase in the stroke animals as compared to the extinction baseline (*q* = 5.55, *p* = 0.0030) and as compared to the sham group (*q* = 3.58, *p* = 0.021). These results suggest that stroke increased the likelihood and intensity of relapse.

In summary, we found that stroke increased alcohol preference from day 5 onwards in rats that were given access to alcohol and water. Additionally, stroke increased operant alcohol self-administration from day 35 onwards. Importantly, stroke also increased the propensity for, and degree of, alcohol relapse.

### Stroke increases the excitability of DMS neurons in alcohol-drinking rats

**Figure 3:**
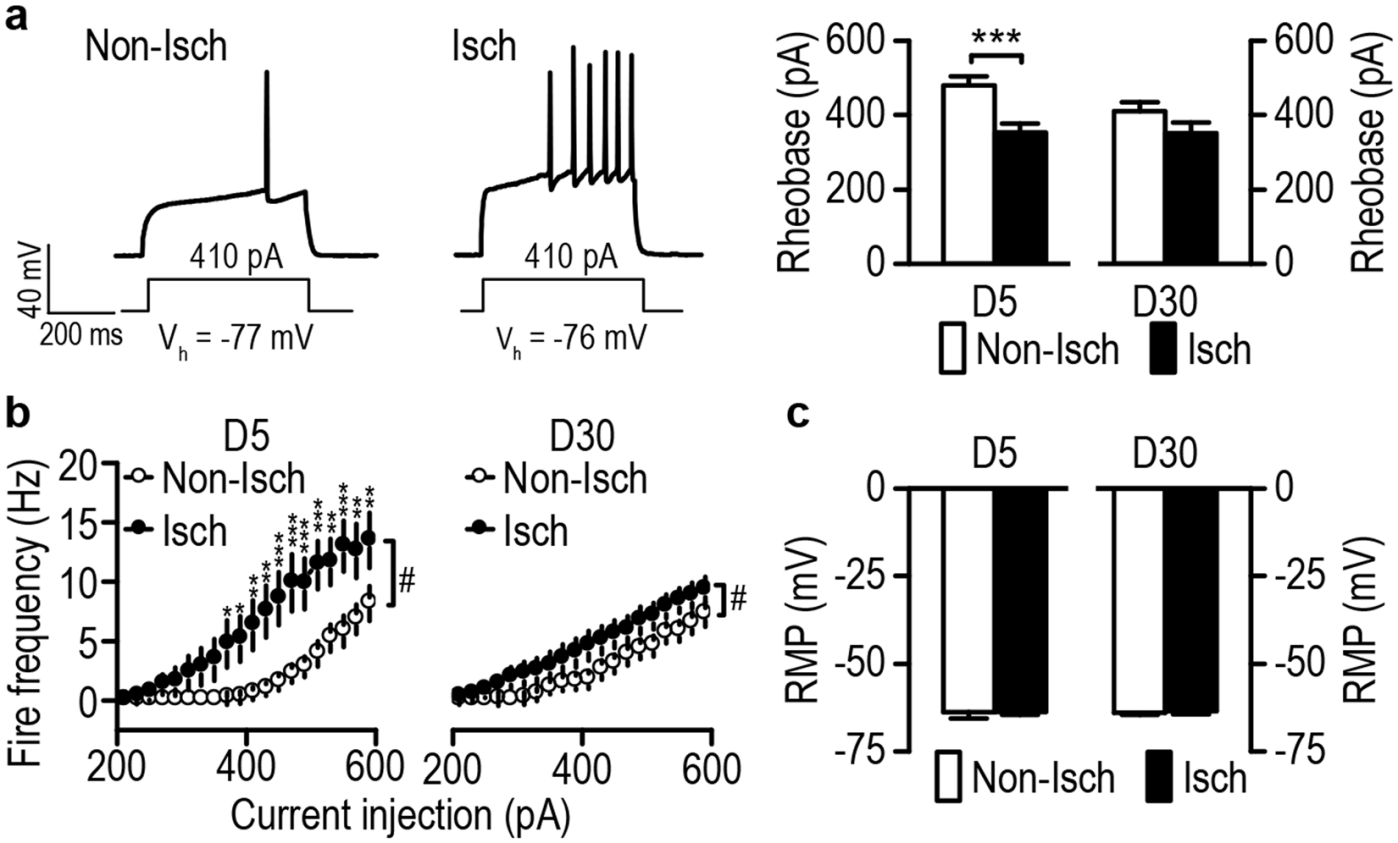
Stroke increases the excitability of DMS neurons in alcohol-drinking rats. Sprague-Dawley rats were trained to consume 20% alcohol for 8 weeks using the intermittent-access 2-bottle choice drinking procedure. Stroke was induced as described in the Experimental Procedures section and the rats were sacrificed on D5 or D30 post-stroke for slice electrophysiological measurement. Evoked action potentials were measured using current-clamp recording in DMS neurons from coronal slices prepared from the non-ischemic (Non-Isch) and ischemic (Isch) hemispheres. (**a**) Stroke reduced the rheobase current in DMS neurons on D5, but not D30. Left and middle, representative DMS action potential traces in response to the same magnitude of injected current on the Non-Isch and Isch sides on D5. Right, bar graphs showing a lower rheobase current on the Non-Isch side than on the Isch side on D5, but not D30. ***p < 0.0001 by *t* test. n = 12 neurons from 3 rats (Non-Isch) and 12 neurons from 4 rats (Isch) for both D5 and D30 groups. (**b**) Stroke increased the evoked firing frequency in DMS neurons on D5 (left) and D30 (right). ^#^ *p* < 0.05 by two-way RM ANOVA; *p < 0.05, **p < 0.01, and ***p < 0.001 by post-hoc SNK test. D5: n = 12 neurons from 3 rats per group; D30: n = 12 neurons from 3 rats (Non-Isch) and 12 neurons from 4 rats (Isch). (**c**) Bar graphs showing no difference in the resting membrane potentials (RMP) on the Non-Isch and Isch sides on D5 or D30. n = 12 neurons from 3 rats (Non-Isch) and 12 neurons from 4 rats (Isch) for both D5 and D30 groups.

Having shown that stroke increased alcohol preference, seeking, and relapse, we next explored the possible mechanisms underlying these changes. The ischemic stroke induced in the present study caused lesions in the DLS, but not in the DMS^20^ (Fig. 1a). The DMS has been reported to control alcohol consumption^14, 15, 19, 27^ and we therefore examined the effect of stroke on neuronal excitability in this region. Animals were trained to drink alcohol using the 2-bottle choice procedure and stroke was induced, as described above. Since ischemic stroke was induced unilaterally on the left side of the brain, we used the right side of the DMS as the non-ischemic control. We conducted whole-cell recording of DMS neurons in striatal slices from stroked rats. As shown in Fig. 3a (left and middle), injection of the same magnitude of current in DMS neurons on day 5 post-stroke induced one spike on the non-ischemic side and 6 spikes on the ischemic side, suggesting stroke may increase the excitability of DMS neurons. Neuronal excitability can be measured with rheobase currents, and we found that stroke had significant effects on the rheobase current (Fig. 3a right). At day 5 there was a significant effect of stroke (*t*_(22)_ = 3.82, *p* = 0.0009) and we found that this current was significantly lower on the ischemic side than on the non-ischemic side, indicating that the DMS neurons on the ischemic side were more excitable than those on the non-ischemic side. However, this effect was not observed on day 30 (Fig. 3a right; *t*_(22)_ = 1.54, *p* = 0.14). Furthermore, as depicted in Fig. 3b, we found a significant effect of stroke (*F*_(1,418)_ = 7.56, *p* = 0.012), stimulation intensity (*F*_(19,418)_ = 40.04, *p* < 0.000001) and stroke x stimulation intensity interaction (*F*_(19,418)_ = 5.39, *p* < 0.000001) on day 5. The frequency of evoked firing of DMS neurons was dramatically higher on post-stroke day 5 (Fig. 3b left; 370 pA: *q* = 3.33, *p* = 0.023; 390 pA: *q* = 3.57, *p* = 0.015; 410 pA: *q* = 4.18, *p* = 0.0050; 430 pA: *q* = 4.78, *p* = 0.0016; 450 pA: *q* = 5.15, *p* = 0.00081; 470 pA: *q* = 5.57, *p* = 0.00038; 490 pA: *q* = 5.09, *p* = 0.0009; 510 pA: *q* = 5.45, *p* = 0.00046; 530 pA: *q* = 4.66, *p* = 0.0020; 550 pA: *q* = 5.21, *p* = 0.00071; 570 pA: *q* = 4.18, *p* = 0.0050; 590 pA: *q* = 3.88, *p* = 0.0088). In addition, we found a significant effect of stroke (*F*_(1,417)_ = 5.23, *p* = 0.032) and stimulation (*F*_(19,417)_ = 35.50, *p* < 0.000001) but no interaction effect (*F*_(19,417)_ = 0.63, *p* = 0.89) on day 30, and the frequency was slightly but significantly higher on day 30 (Fig. 3b right) on the ischemic side, as compared to the non-ischemic side (*q* = 3.23 *p* = 0.03). Lastly, as depicted in Fig. 3c, the resting membrane potential was similar on days 5 (Mann-Whitney U = 59.00, *p* = 0.47) and 30 (Mann-Whitney U = 66.5, *p* = 0.77) post-stroke. Together, these results suggest that ischemic stroke increases neuronal excitability without changing the resting membrane potential of DMS neurons in alcohol-drinking animals.

### Stroke enhances glutamatergic inputs onto DMS D1-neurons in alcohol-drinking rats

**Figure 4:**
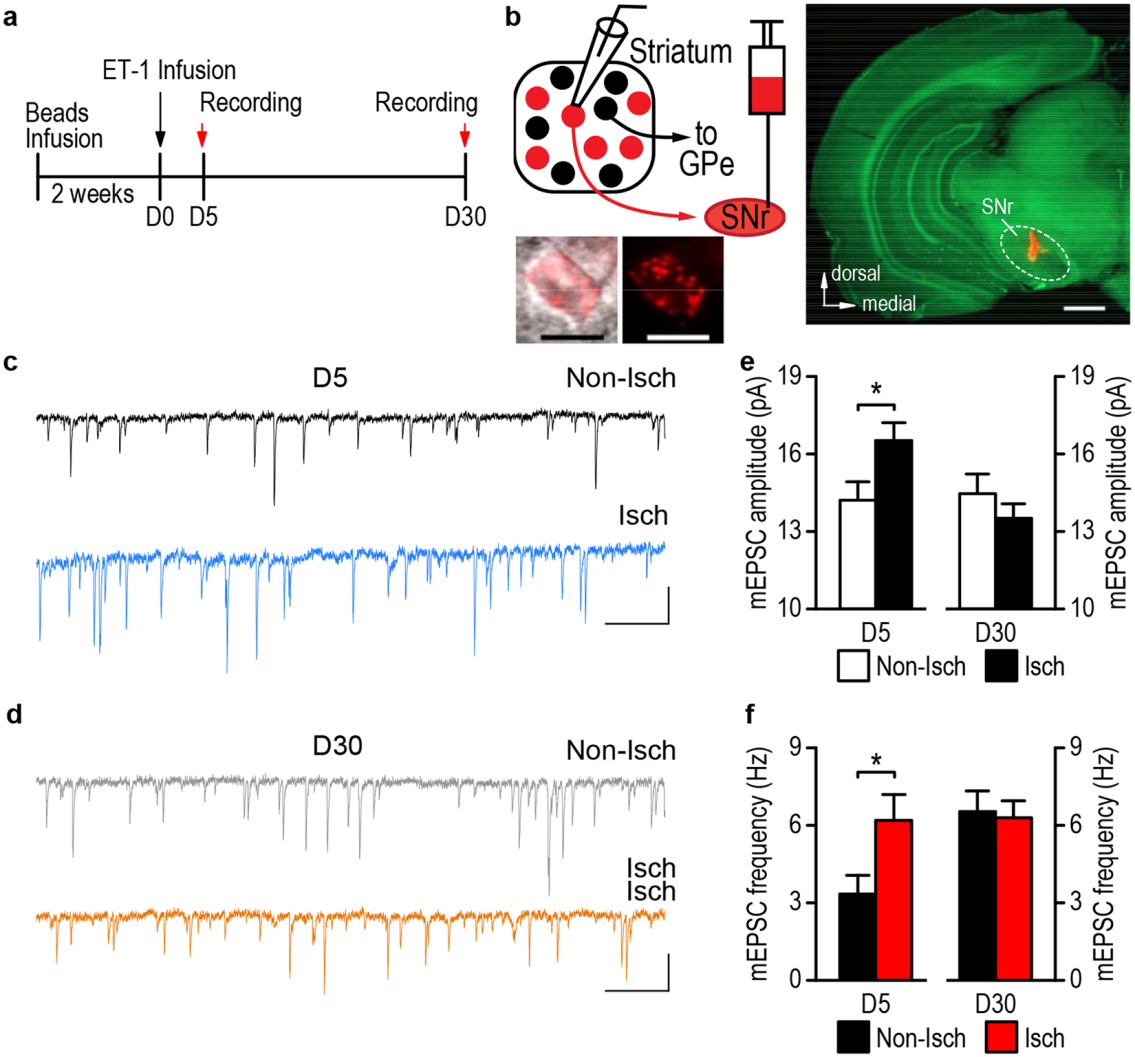
Stroke enhances glutamatergic drive of DMS D1-MSNs in alcohol-drinking rats. (**a**) Schematic illustration of the experimental procedure. (**b**) Retrograde labeling of D1-MSNs. Left top, diagram showing that retrograde beads infused into the SNr labeled DMS D1-MSNs. Right, representative image depicting the infusion site in the SNr. Scale bar: 1 mm. Left bottom, sample image of bead-labeled D1-MSNs. Scale bar: 10 µm. (**c** and **d**) Representative mEPSC traces from the non-ischemic (Non-Isch) and ischemic (Isch) sides on day 5 (D5, **c**) and day 30 (D30, **d**). Scale bars: 0.5 s, 10 pA. (**e**) Bar graphs showing an increased mEPSC amplitude on the Isch side, as compared to the Non-Isch side on D5 (left), and no differences between the two groups on D30 (right). *p < 0.05 by *t* test. D5: n = 11 (Non-Isch) and 17 (Isch) neurons from 7 rats per group; D30: n = 13 (Non-Isch) and 11 (Isch) neurons from 7 rats per group. (**f**) Bar graphs depicting an increase in mEPSC frequency on the Isch side, as compared to the Non-Isch side on D5 (left), and no differences between these two groups on D30 (right). *p < 0.05 by *t* test. D5: n = 11 (Non-Isch) and 17 (Isch) neurons from 7 rats per group; D30: n = 13 (Non-Isch) and 11 (Isch) neurons from 7 rats per group.

Addictive behaviors in drug and alcohol abuse are influenced not only by altered intrinsic excitability but also by aberrant glutamatergic plasticity in specific medium spiny neurons (MSNs) that express dopamine D1 receptors^14, 15, 17^. Therefore, we examined whether glutamatergic transmission onto D1-MSNs was altered after stroke. Rats were trained to drink alcohol using the 2-bottle choice procedure described above. We infused retrograde beads into the SNr two weeks prior to stroke induction (Figs 4a and 4b). This caused selective labeling of striatonigral MSNs (Fig. 4b) because these, unlike other striatal neurons, project to the SNr^14, 15, 28^. It was reported that 92% of these retrogradely labeled striatonigral MSNs contained D1 receptors and did not overlap with D2-MSNs^29, 30^, we thus consider bead-positive neurons as D1-MSNs. We found that the amplitudes of AMPA receptor (AMPAR)-mediated miniature excitatory postsynaptic currents (mEPSCs) were increased in D1-MSNs on the ischemic side on post-stroke day 5 (Figs 4c and 4e left; *t*_(26)_ = −2.24, *p* = 0.030), but not day 30 (Figs 4d and 4e right; *t*_(22)_ = 1.54, *p* = 0.14), as compared to the non-ischemic side. We also observed a higher frequency on the ischemic side than on the non-ischemic side on post-stroke day 5 (Figs 4c and 4f left; *t*_(26)_ = −2.07, *p* = 0.048), but not day 30 (Figs 4d and 4f right; *t*_(22)_ = 0.23, *p* = 0.82). Taken together, these results suggest that stroke increases the strength of glutamatergic inputs onto DMS D1-neurons on the ischemic side.

### Stroke increases the spontaneous firing of dopaminergic neurons in the SNc of alcohol-drinking rats

**Figure 5:**
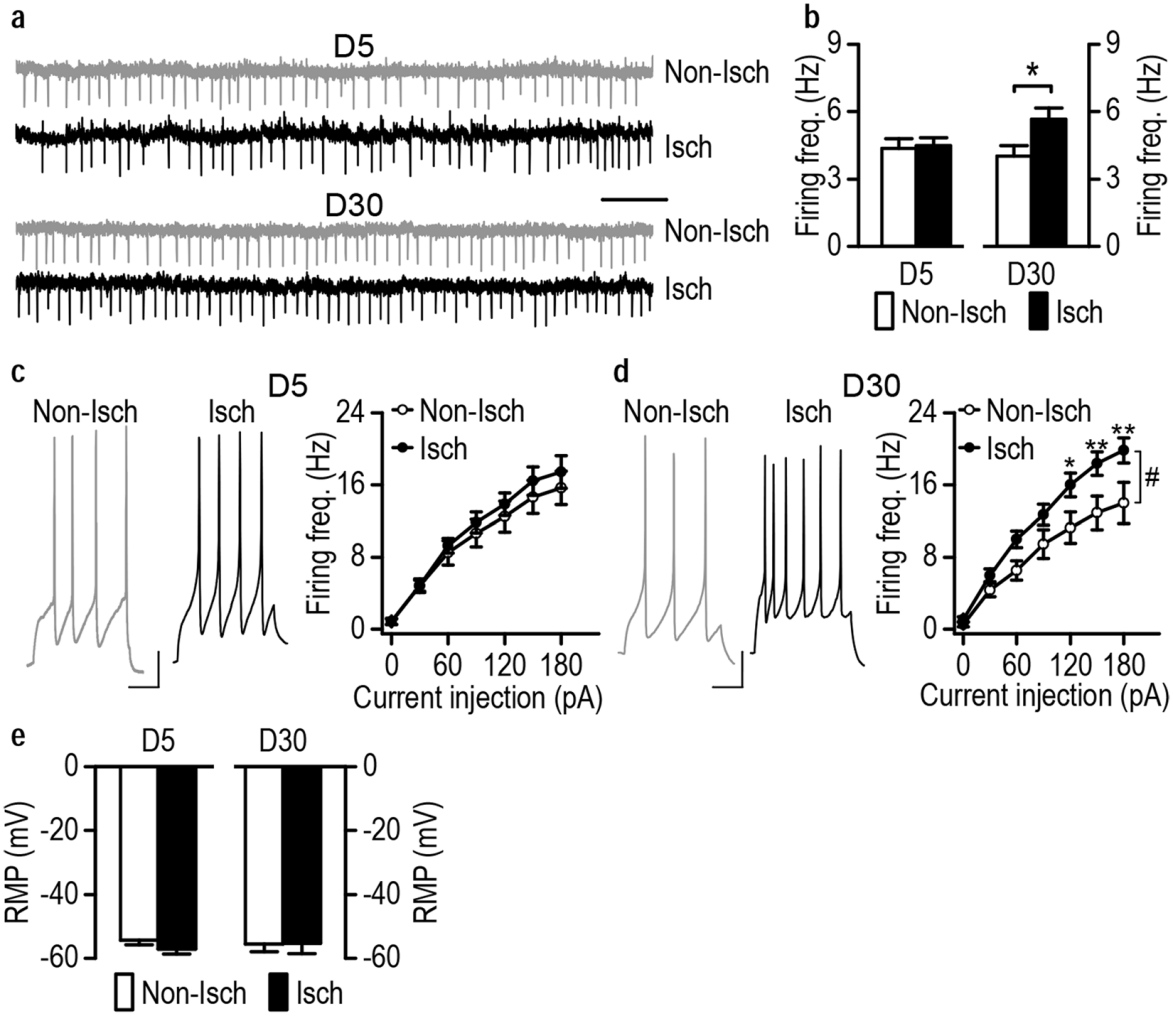
Stroke enhances spike firing activity of SNc neurons in alcohol-drinking rats. Rats were trained to consume alcohol using the 2-bottle choice procedure and stroke was induced as above. Midbrain slices were prepared on D5 and D30 post-stroke, and firing activity of SNc neurons were measured. (**a**) Sample traces of spontaneous firing of SNc neurons on the ipsilesional ischemic (Isch) and contralesional non-ischemic (Non-Isch) sides on D5 and D30. (**b**) Bar graph showing similar spontaneous firing frequencies on D5 (left) and increased spontaneous firing frequency on the Isch side on D30 (right), as compared to the Non-Isch side. *p < 0.05, *t* test. D5: n = 9 (Non-Isch) and 12 (Isch) neurons from 6 rats per group; D30: n = 12 neurons from 7 rats (Non-Isch) and 10 neurons from 6 rats (Isch). (**c**) Stroke did not change SNc neuron excitability on D5. Sample traces of membrane potentials following two 500-ms current injections are shown for the Non-Isch (left) and Isch (middle) sides. Right, relationship between the injected current magnitude and firing frequency of SNc neurons on both sides. n = 12 (Non-Isch) and 14 (Isch) neurons from 7 rats per group. (**d**) Stroke produced an increase in excitability on D30. Sample traces of membrane potentials following two 500-ms current injections are shown for the Non-Isch (left) and Isch (middle) sides. Right, relationship between the injected current magnitude and firing frequency of SNc neurons on both sides. ^#^ *p* < 0.05 by two-way RM ANOVA; *p < 0.05, **p < 0.001, and ^#^ *p* < 0.05 by post-hoc SNK test. n = 11 neurons from 7 rats (Non-Isch) and 11 neurons from 8 rats (Isch). (**e**) Bar graphs showing no difference between the resting membrane potentials (RMP) on the Non-Isch and Isch sides on D5 (left) or D30 (right). D5: n = 12 neurons from 5 rats (Non-Isch) and 14 neurons from 6 rats (Isch); D30: n = 11 neurons from 7 rats (Non-Isch) and 11 neurons from 8 rats (Isch). Scale bars: 1 s (**a**) and 150 ms, 10 mV (**c**,**d**).

Stroke-induced lesion of the DLS may cause secondary remote changes in the midbrain dopaminergic neurons that project to the dorsal striatum. To examine this possibility, SD rats were trained to drink alcohol using the 2-bottle choice procedure and stroke was induced, as described above. The spontaneous firing rates of SNc neurons were measured on days 5 and 30 post-stroke. We found that on post-stroke day 5, the firing frequencies were similar in both hemispheres (Fig. 5a and b left; *t*_(19)_ = −0.23, *p* = 0.82). However, on post-stroke day 30, the spontaneous firing frequency was significantly higher in the ipsilesional midbrain than in the contralesional midbrain (Figs 5a and 5b right; Mann-Whitney U = 23, *p* = 0.016), suggesting that a secondary change in the SNc had occurred between days 5 and 30. Similarly, the frequency of evoked firing was unaltered on day 5 (Fig. 5c; stroke: *F*_(6,144)_ = 0.41, *p* = 0.53), but had significantly increased by day 30 post-stroke (Fig. 5d; stroke: *F*_(1,120)_ = 4.82, *p* = 0.040; stimulation: *F*_(6,120)_ = 138.55, *p* < 0.00001; stroke x stimulation interaction *F*_(6,120)_ = 3.89, *p* = 0.0014) in the ipsilesional midbrain, as compared to the contralesional midbrain at 120, 150 and 180 pA (*q* = 3.59, *p* = 0.016; *q* = 4.14, *p* = 0.0061; and *q* = 4.42, *p* = 0.0038 respectively). The resting membrane potentials did not differ on either side of the midbrain after ischemic stroke (Fig. 5e; D5: *t*_(20)_ = 1.60, *p* = 0.13; D30: *t*_(24)_ = −0.08, *p* = 0.47).

**Figure 6:**
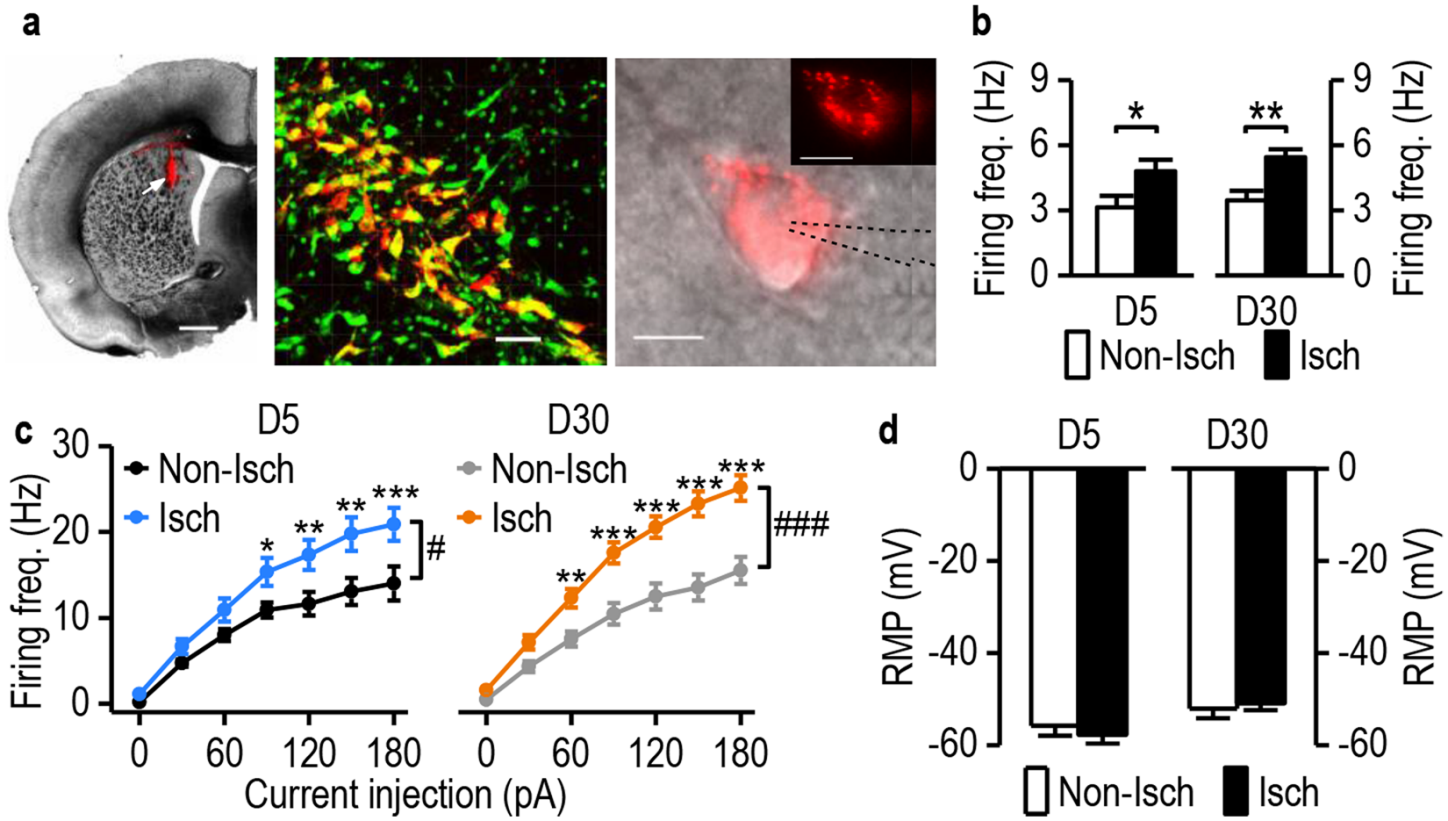
Stroke increases spike firing frequency of DMS-projecting SNc neurons in alcohol-drinking rats. (**a**) Retrograde labeling of DMS-projecting SNc neurons. Left, Light microscope image showing the retrograde red beads (arrow) that were injected into the DMS. Middle, confocal image of the SNc showing bead-positive neurons (yellow). Sections were stained with NeuroTrace green. Right, differential interference contrast (DIC) and red-fluorescent image of a recorded DMS-projecting SNc neuron. Scale bars: 1 mm (left), 50 µm (middle), 10 µm (right), 10 µm (right, inset). (**b**) Bar graphs showing increased spontaneous firing frequencies on D5 (left) and D30 (right). *p < 0.05 and **p < 0.01 by *t* test. D5: n = 9 neurons from 6 rats (Non-Isch) and 11 neurons from 7 rats (Isch); D30: n = 12 neurons from 7 rats (Non-Isch) and 11 neurons from 9 rats (Isch). (**c**) Stroke increased the excitability of DMS-projecting SNc neurons on D5 (left) and D30 (right). ^#^ *p* < 0.05 and ^###^ *p* < 0.001, two-way RM ANOVA, *p < 0.05, **p < 0.01, and ***p < 0.001 vs. Non-Isch, with the same intensity of current injection, post-hoc SNK test. D5: n = 11 neurons from 6 rats (Non-Isch) and 9 neurons from 8 rats (Isch); D30: n = 13 neurons from 9 rats (Non-Isch) and 14 neurons from 8 rats (Isch). (**d**) Bar graphs showing no difference between the resting membrane potentials (RMP) on the Non-Isch and Isch sides, although an overall difference between D5 (left) and D30 (right) was found. D5: n = 11 neurons from 7 rats (Non-Isch) and 9 neurons from 8 rats (Isch); D30: n = 13 neurons from 9 rats (Non-Isch) and 14 neurons from 8 rats (Isch).

It has been reported that more than 95% of DMS-projecting SNc neurons are dopaminergic^25^. To determine whether these SNc neurons were specifically affected, we infused retrograde beads into the DMS two weeks prior to stroke surgery in a separate group of rats (Fig. 6a). We found a significant effect of stroke as early as day 5, in that the spontaneous firing rates in DMS-projecting dopaminergic SNc neurons were higher in the ipsilesional midbrain than in the equivalent contralesional region (Fig. 6b left; *t*_(18)_ = −2.19, *p* = 0.042), which was still observed on day 30 (Fig. 6b right; Mann-Whitney U = 14.00, *p* = 0.0015). When we looked at the frequency of evoked firing on day 5, there was an effect of stroke (Fig. 6c left; *F*_(1,108)_ = 7.74, *p* = 0.012), stimulation intensity (*F*_(6,108)_ = 98.70, *p* < 0.00001) and a stroke x intensity interaction effect (*F*_(6,108)_ = 3.64, *p* = 0.0025). Post-hoc analysis revealed a significant increase in the ipsilesional midbrain at multiple stimulation intensities on day 5 (Fig. 6c left; 90 pA: *q* = 3.30, *p* = 0.025; 120 pA: *q* = 4.25, *p* = 0.0046; 150 pA: *q* = 4.99, *p* = 0.0012; 180 pA: *q* = 5.14, *p* = 0.00059) and on day 30 post-stroke we identified similar significance (Fig. 6c right; stroke: *F*_(1,150)_ = 19.05, *p* = 0.00019; stimulation intensity: *F*_(6,150)_ = 223.47, *p* < 0.00001; stroke x intensity interaction: *F*_(6,150)_ = 12.70, *p* < 0.000001). Furthermore, we identified a significant increase in the ipsilesional midbrain at multiple stimulation intensities on day 30 (Fig. 6c right; 60 pA: *q* = 4.03, *p* = 0.0066; 90 pA: *q* = 6.04, *p* = 0.00021; 120 pA: *q* = 6.89, *p* = 0.00013; 150 pA: *q* = 8.28, *p* = 0.00012; 180 pA: *q* = 8.16, *p* = 0.00012). The resting membrane potentials remained unchanged in the SNc of both hemispheres across the post-stroke test period (Fig. 6d; stroke: *F*_(1,43)_ = 0.05, *p* = 0.83), although there was an overall difference between D5 and D30 (day: *F*_(1,43)_ = 7.13, *p* = 0.011). Then, we measured firing activity of bead-negative neurons, which contains non-DMS-projecting one. Interestingly, we found that the rates of spontaneous (Supplementary Fig. S2a, S2b; *t*_(16)_ = 2.18, *p* = 0.044) and evoked (Supplementary Fig. S2c; *F*_(1,125)_ = 10.49, *p* = 0.004) spike firing were lower in the ipsilesional than contralesional SNc neurons without a change in their resting membrane potentials (Supplementary Fig. S2d). Taken together, these results indicate that stroke causes an immediate and long-lasting increase in the firing activity of DMS-projecting dopaminergic neurons within the SNc.

### Stroke disinhibits DMS-projecting dopaminergic SNc neurons

**Figure 7:**
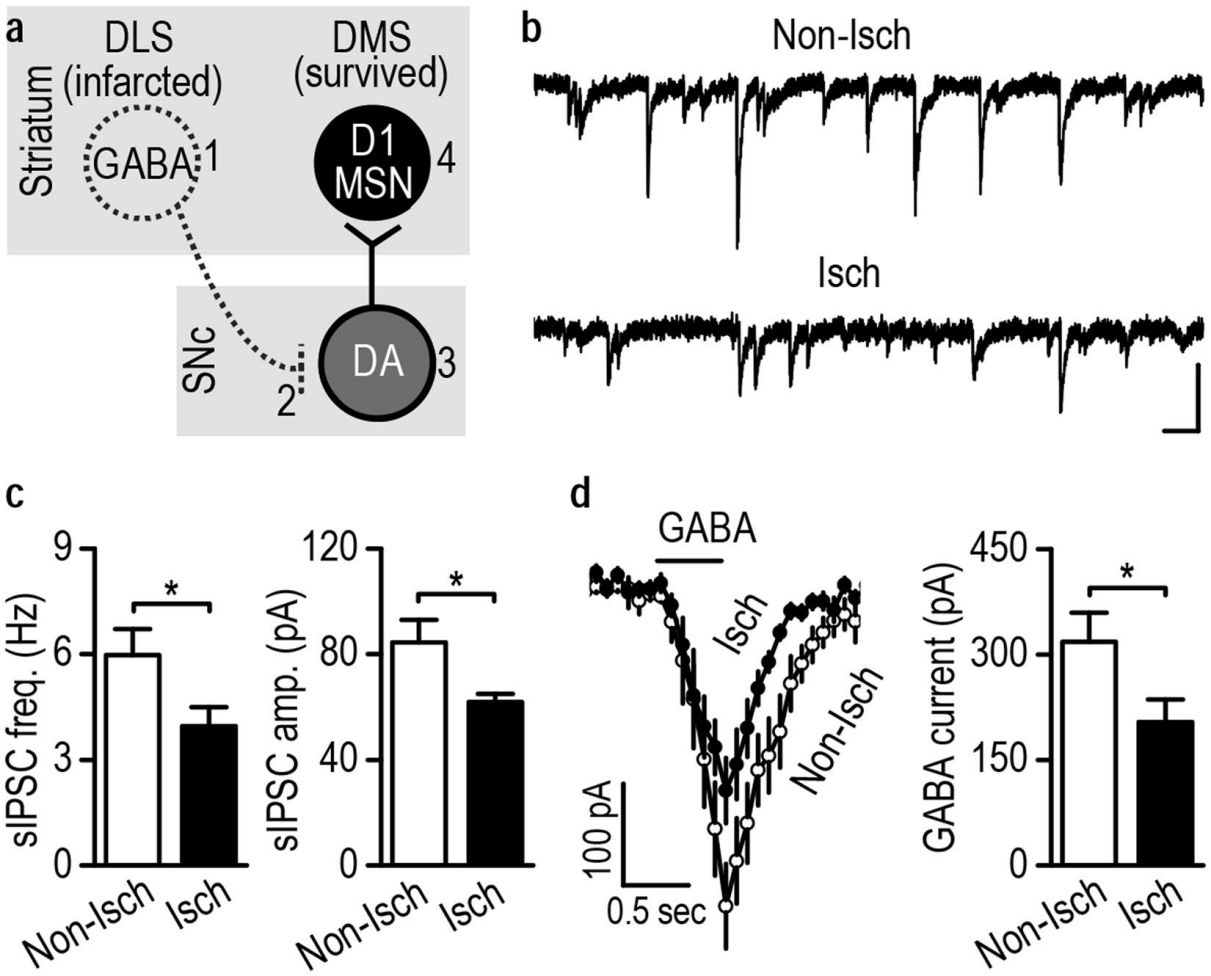
Stroke-mediated DLS infarction reduces GABAergic inputs to DMS-projecting SNc neurons. (*a*) Schematic of proposed changes in striatal and midbrain neurons after DLS infarction. The death of GABAergic DLS neurons (1) may disinhibit dopamine neurons (2) that project to the DMS, leading to increased activity of these neurons (3, 4). (**b**) Representative sIPSC traces in DMS-projecting SNc neurons on the Non-Isch and Isch sides. Scale bars: 50 ms, 100 pA. (**c**) Stroke-induced DLS infarction reduced the frequency and amplitude of sIPSCs in DMS-projecting SNc neurons. Left, bar graphs summarizing the average sIPSC frequencies. *p < 0.05. Right, bar graphs displaying the average amplitudes. *p < 0.05, *t* test. n = 12 neurons from 4 rats (Non-Isch) and 12 neurons from 5 rats (Isch) for both left and right graphs. (**d**) Stroke-induced DLS infarction resulted in lower GABA-induced currents in DMS-projecting SNc neurons. Left, changes in the holding current in DMS-projecting SNc neurons on the contralesional non-ischemic (Non-Isch) and ipsilateral ischemic (Isch) sides. Right, bar graphs summarizing the averaged GABA currents in SNc neurons on both sides. *p < 0.05, *t* test. n = 9 neurons from 5 rats per group.

How does stroke increase the firing activity of DMS-projecting dopaminergic neurons in the SNc? A recent elegant study suggests that the DLS provides the major input (~28% of all inputs) to DMS-projecting dopaminergic neurons, forming the DLS-SNc-DMS (striato-nigro-striatal) circuit^25^ (Fig. 7a). Since the striatal projection neurons are GABAergic, stroke-induced neuronal death in the DLS may disinhibit DMS-projecting neurons in the SNc, thus increasing their firing activity (Fig. 7a). To test this possibility, we measured the GABAergic activity of DMS-projecting SNc neurons on 5 and 30 days post-stroke. We found that on D5, the frequency but not the amplitude of spontaneous inhibitory postsynaptic currents (sIPSCs) was lower in the ipsilesional than contralesional DMS-projecting SNc neurons (Supplementary Fig. S3; frequency: *t*_(24)_ = 8.62; *p* = 0.000; amplitude: *t*_(24)_ = 1.18, *p* = 0.25). Interestingly, we discovered that on D30, both the frequency and the amplitude of the sIPSC were lower in the ipsilesional SNc than in the contralesional SNc (Figs 7b and 7c; frequency: *t*_(22)_ = 2.23; *p* = 0.036; amplitude: *t*_(22)_ = 2.49, *p* = 0.021). The reduced sIPSC amplitude indicates a postsynaptic change in GABAergic receptor responsiveness. Thus, we measured GABA-induced currents on D30 and found lower GABA currents in the ipsilesional midbrain than in the equivalent contralesional region (Fig. 7d; *t*_(16)_ = 2.16, *p* = 0.046). Together, these results suggest that stroke induced immediate and persistent reduction in the strength of GABAergic inputs onto DMS-projecting dopaminergic SNc neurons.

### Inhibition of D1 receptors attenuates operant self-administration of alcohol

**Figure 8:**
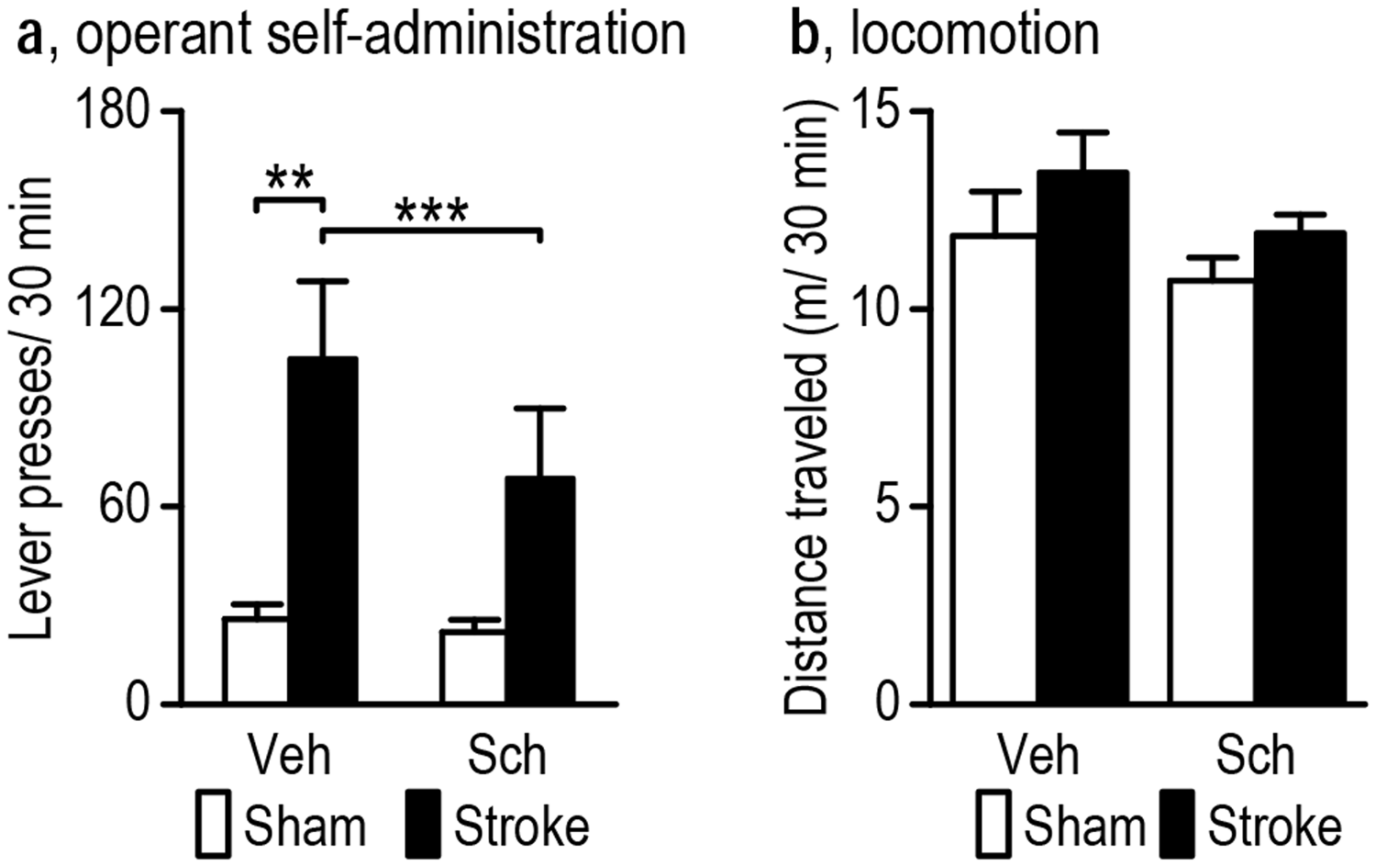
D1 receptor inhibition attenuates the stroke-induced increase in operant alcohol self-administration. Sprague-Dawley rats were administered an intraperitoneal injection of saline (Veh, 1 ml/kg) or SCH 23390 (SCH, 1 µg/ml/kg) 30 min prior to the operant self-administration session. (**a**) D1 receptor inhibition by SCH did not affect operant lever presses in the sham group and significantly decreased operant lever presses in the stroke group, as compared to vehicle-treated rats. **p < 0.01 and ***p < 0.001, two-way RM ANOVA followed by the post-hoc SNK test. n = 6 (Sham) and 6 (Stroke) rats. (**b**) D1 receptor inhibition by SCH had no effect on locomotion in either the stroke or sham group. n = 6 (Sham) and 5 (Stroke) rats.

Based on our current observation of stroke-mediated enhancement of DMS-projecting dopaminergic activity and glutamatergic strength onto DMS D1-neurons, and our previous finding that D1 receptor inhibition in the DMS reduced alcohol intake^15^, we examined whether the stroke-induced increase in alcohol-seeking was blocked by D1 receptor inhibition. Rats were allowed to self-administer alcohol in an operant setting, as shown in Fig. 2. Thirty minutes prior to their testing sessions, the animals received an intraperitoneal injection of vehicle (1 ml/kg saline) or SCH 23390 (1 µg/ml/kg; Fig. 8a). The testing was conduced on D65 post-stroke, which was between D56 and D70, a time period showing a reliable increase in operant self-administration (Fig. 2a). We discovered a significant effect of stroke (*F*_(1,9)_ = 9.09, *p* = 0.015), drug treatment (*F*_(1,9)_ = 55.94, *p* < 0.00001) and an interaction effect (*F*_(1,9)_ = 35.92, *p* = 0.00020). We found that the vehicle-treated stroke group executed significantly more lever presses than the vehicle-treated sham control animals (*q* = 5.32, *p* = 0.0044) and that D1 inhibition significantly decreased lever presses in the stroke group (*q* = 12.90, *p* = 0.00021) but had no effect on lever presses in the sham group (*q* = 1.56, *p* = 0.30). Furthermore, D1 inhibition had no effect on locomotion in the stroke or sham groups (Fig. 8b; *F*_(1,8)_ = 4.41, *p* = 0.069). Taken together, these data suggest that the sustained stroke-induced increase in alcohol consumption can be blocked by D1 receptor inhibition.

## Discussion

In this study, we discovered that ischemic stroke-induced infarction of the DLS increased voluntary alcohol intake, preference for alcohol, alcohol-seeking, and relapse behaviors in rats. In line with these behavioral changes, we observed that the excitabilities of DMS neurons and dopaminergic SNc neurons were increased after stroke. Furthermore, glutamatergic drive of DMS D1-neurons was potentiated and GABAergic drive of SNc neurons was weakened following stroke induction. Importantly, systemic inhibition of D1 receptors attenuated these ischemic stroke-induced alcohol-seeking behaviors, implying a causal relationship between the observed neuroplasticity and the behavioral changes. These findings indicate that stroke triggers aberrant neuroplasticity in the nigrostriatal circuit and this enhances alcohol-related addictive behaviors during recovery. Prevention of these behaviors may help to reduce the risk for stroke recurrence.

The most important finding of this research was the novel demonstration that ischemic stroke-induced infarction of the DLS in an animal model increased home-cage alcohol preference, alcohol-seeking, and relapse. Interestingly, we found that when alcohol and water were both provided, alcohol preference was increased as early as day 5 after the ischemic stroke. The increased preference results from the decreased water consumption, and the decrease may contribute to the body-weight change on D5. Importantly, the changes in preference may reflect an increased motivation for alcohol. Lever presses for alcohol in an operant self-administration test began to increase on post-stroke day 35 and this change was sustained until at least day 70 post-stroke, suggesting that stroke increased the alcohol-seeking motivation of these rats. These results were consistent with human studies indicating that patients with stroke showed increased alcohol consumption^31–33^. Collectively, these results provide an invaluable insight into clinical observations suggesting that alcohol-addicted patients have a high risk of developing an increased motivation for alcohol and heightened risk for relapse to alcohol consumption after stroke.

The present study found that the increased post-stroke alcohol preference, consumption and relapse were accompanied by an enhancement of intrinsic excitability and glutamatergic transmission in the DMS. Given that alcohol preference is positively regulated by increased excitability of striatal neurons^34^ and by chemogenetic excitation of specific D1-MSNs^14^, it is likely that stroke increased D1-MSN excitability leading to increased alcohol preference on D5 post-stroke. We used retrograde beads to identify the specific striatonigral pathway, facilitating measurement of synaptic transmission in D1-neurons within the DMS. We found that ischemic stroke increased AMPAR-mediated glutamatergic strength onto D1-neurons, consistent with our recent study showing that alcohol consumption increased synaptic AMPAR activity in D1-neurons^15^. Furthermore, chemogenetic mimicking of their glutamatergic activation increased alcohol intake^15^. Taken together, these data suggest that ischemic stroke-mediated potentiation of AMPAR activity in DMS D1-neurons may account for the increases in alcohol preference.

The dopaminergic neurons innervating the DMS are located in the SNc^12^. Unlike the DMS neurons, which showed increased excitability on day 5 post-stroke, dopaminergic SNc neurons changed their spontaneous and evoked firing activities starting on day 30 post-stroke. These findings were consistent with a previous study showing that infarction of the striatum increased the excitability of SNc neurons^35^. However, when the specific DMS-projecting dopaminergic SNc neurons were recorded, we observed an augmentation in their spontaneous and evoked firing activities from post-stroke day 5 onwards, suggesting that stroke triggers early changes in the nigrostriatal neural circuit. The augmented activity of specific DMS-projecting neurons and the decreased activity of bead-negative (possibly non-DMS-projecting) neurons are consistent with the finding that the average neuronal activity was not changed. These non-DMS-projecting SNc neurons may express dopamine D2 receptors^23, 36, 37^, through which DMS-projecting neuron-released dopamine inhibits non-DMS-projecting neuronal activity. It is well known that dendritically released dopamine activates D2 receptors leading to inhibition of dopaminergic firing in the midbrain^23, 36, 38, 39^. Furthermore, some non-DMS-projecting SNc neurons, e.g., those DLS-projecting ones, may be degenerated on D30^40–42^ due to stroke-induced reduction of neurotrophic factors^43^ that are normally produced in the striatum and retrogradely transported to the midbrain where they support the survival of dopaminergic neurons^44–46^. This neurodegeneration is predicted to increase the proportion of DMS-projecting to non-DMS-projecting neurons, leading to elevated firing frequency of overall SNc neurons on D30. DMS-projecting SNc neurons also showed reduced GABAergic drive, which may contribute to the increased firing activity of dopaminergic neurons. The reduced amplitudes of GABA-induced currents and sIPSCs on D30 may be adaptive response to the decreased DLS input. Taken together, these findings indicate that ischemic stroke decreases inhibitory GABAergic transmission, resulting in long-lasting enhancement of neuronal excitability in DMS-projecting dopaminergic SNc neurons, which drives alcohol addictive behaviors. Lastly, the current stroke model also lesioned part of the cortex and ventral striatum, which may additionally contribute to the increased alcohol intake post-stroke.

In summary, the present findings demonstrate that the stroke-induced increases in alcohol-seeking and relapse are associated with abnormal intrinsic and synaptic plasticity in nigrostriatal dopaminergic SNc neurons and DMS D1-neurons; the latter have been previously reported to control drug and alcohol intake^15, 47, 48^. This study identified cellular- and circuit-based mechanisms involved in stroke-induced increases in alcohol consumption. We also showed that systemic blockade of D1 receptors that are highly expressed in the DMS D1-neurons^14, 15^ but not in SNc neurons attenuated the stroke-induced increase in alcohol intake, indicating that this approach could provide a clinical approach to limiting alcohol consumption by stroke patients, thus reducing the likelihood of stroke recurrence.

## Methods

### Reagents

Tetrodotoxin (TTX) and AMPA were obtained from Tocris. DNQX (6,7-dinitroquinoxaline-2,3-dione) and CPP (3-[2-carboxypiperazin-4-yl]propyl-1-phosphonic acid) were purchased from Abcam. Red and green fluorescent RetroBeads were obtained from LumaFlour Inc. (Florida). Endothelin-1 was purchased from the American Peptide Company (California). SCH 23390, picrotoxin, and all other chemicals were purchased from Sigma.

### Animals

Male SD rats were purchased from Harlan Laboratory. Rats were housed individually at 23 °C under a 12-h light:dark cycle, with lights on at 7:00 A.M. Food and water were provided *ad libitum*. All animal care and experimental procedures were approved by the Texas A&M University Institutional Animal Care and Use Committee and were conducted in accordance with the National Research Council *Guide for the Care and Use of Laboratory Animals*.

### Ischemic stroke

An endothelin-1-induced middle cerebral artery occlusion model of ischemic stroke^20^ was used. Animals were anesthetized (ketamine-xylazine). Endothelin-1 (2 µl) was infused adjacent to the left middle cerebral artery in the striatum at AP +0.9, ML +3.4, DV −8.5. Sham control animals were injected with saline.

### Intermittent-access to 20% alcohol 2-bottle choice drinking procedure

The procedure was conducted as described previously^19, 27^. Briefly, rats were given 24-h concurrent access to one bottle of 20% alcohol in water and one bottle of water with 24- or 48-h periods of alcohol-deprivation. Alcohol intake (g/kg/d) was measured as the weight of 20% alcohol solution consumed x 20% (Fig. 1c) and was normalized by dividing it by the ratio of locomotion at the same time point to the baseline locomotion (Fig. 1h). Alcohol preference was calculated as the percentage of 20% alcohol solution consumed relative to total fluid intake (20% alcohol + water).

### Operant self-administration of alcohol

#### Measurement of alcohol seeking and intake

SD rats were trained to self-administer a 20% alcohol solution in an operant chamber^19, 26, 27^. The chamber contained two levers; active lever for solution delivery, and inactive lever that could be pressed and recorded but did not deliver anything. After a 48-h exposure to 20% alcohol in the home cage and one overnight session in an operant chamber where the active lever delivered 0.1 ml of water in a fixed ratio of 1 (FR1), operant sessions were conducted for 5 days per week for 2 weeks using the FR1 schedule. These sessions initially lasted 3 h and were shortened 30 min, with an active lever press resulted in delivery of 0.1 ml of 20% alcohol. During the third week, operant sessions were run 3 days/week and the schedule was increased to FR3. Once stabilized, stroke was induced as described above. Alcohol seeking and intake were monitored for 70 days with extinction and reinstatement testing. Alcohol seeking was measured using the number of lever presses and alcohol intake was determined by subtracting the weight of the alcohol after the 30-min operant session from the weight prior to the session. That weight was divided by the kg weight of the animal (g/kg).

#### Measurement of alcohol extinction and relapse

To extinguish the behavior, operant sessions were conducted daily in which responses were recorded but no alcohol delivered. After a minimum of 9 extinction sessions, the average number of lever presses across 3 consecutive sessions was <10^27^, reinstatement was conducted with or without D1 receptor inhibition. Reinstatement was induced by the non-contingent delivery of 0.1 ml of 20% alcohol into the reward port immediately at the beginning of the session^27^. During the remainder of the 30-min session, subsequent presses on the active lever were recorded but no alcohol delivered. The experiment was conducted using a counterbalanced within-subject design, resulting in 2 tests per rat. Reinstatement tests were followed by 1 week of reacquisition of alcohol self-administration, and followed by another extinction period and reinstatement test. To test the effect of a D1 antagonist on this behavior, rats were administered an intraperitoneal injection of vehicle (1 ml/kg saline) or SCH 23390 (1 µg/ml/kg) 30 min prior to the operant self-administration session.

### Retrograde labeling of D1-MSNs and DMS-projecting SNc neurons

Animals were anesthetized with isoflurane. Fluorescent RetroBeads were bilaterally infused into the SNr (AP −5.28 mm, ML ±2.0 mm, DV −8.3 mm) and two DMS sites (AP 1.2 mm, ML ±1.8 mm, DV −4.8 mm; AP 0.38 mm, ML ±2.3 mm, DV −4.8 mm) in order to label SNr-projecting DMS neurons, which are presumably dopamine D1 receptor-expressing medium spiny neurons (D1-MSNs)^14, 15, 24, 28^, and DMS-projecting dopaminergic SNc neurons, respectively. After surgery, all animals were allowed to recover for 3 days before resuming alcohol-drinking procedure.

### Open field locomotion test

Each animal was placed in the center of an open field chamber and allowed to freely explore the apparatus for 30 min. The open field consisted of a square arena (16 cm × 16 cm) and the rat movements were tracked by 15 laser beam sensors on each x- and y-axis^14^. To test the effect of a D1 antagonist, rats received an intraperitoneal injection of vehicle (1 ml/kg saline) or SCH 23390 (1 µg/ml/kg) 30 min prior to the open field session. For each animal, the total distance traveled during the 30-min test period was calculated.

### Electrophysiology

Twenty-four hours after the completion of the last alcohol-drinking session, animals were sacrificed and 250-µm coronal sections containing the DMS (Figs 4 and 5) or 150-µm horizontal sections containing the SNc (Figs 6–8) were prepared in an ice-cold cutting solution (in mM)^27, 44^: 40 NaCl, 148.5 sucrose, 4 KCl, 1.25 NaH_2_PO_4_, 25 NaHCO_3_, 0.5 CaCl_2_, 7 MgCl_2_, 10 glucose, 1 sodium ascorbate, 3 sodium pyruvate, and 3 myoinositol, saturated with 95% O_2_ and 5% CO_2_. Slices were then incubated in a 1:1 mixture of cutting solution and external solution at 32 °C for 45 min. The external solution contained the following (in mM): 125 NaCl, 4.5 KCl, 2.5 CaCl_2_, 1.3 MgCl_2_, 1.25 NaH_2_PO_4_, 25 NaHCO_3_, 15 sucrose, and 15 glucose, saturated with 95% O_2_ and 5% CO_2_. Slices were then maintained in external solution at room temperature until use.

Slices were perfused with the external solution at a flow rate of 3–4 ml/min. D1-neurons in the DMS or DMS-projecting neurons in the SNc were identified by the red RetroBead fluorescence. Whole-cell patch-clamp and cell-attached recordings were made using a MultiClamp 700B amplifier with Clampex 10.4 software (Molecular Devices). The pipette solution contained (in mM): 119 CsMeSO_4_, 8 TEA.Cl, 15 HEPES, 0.6 EGTA, 0.3 Na_3_GTP, 4 MgATP, 5 QX-314.Cl, and 7 Na_2_CrPO_4_. For voltage-clamp recording, striatal neurons were clamped at −70 mV and SNc neurons were clamped at −60 mV. For current-clamp recording, the electrode contained (in mM): 123 potassium gluconate, 10 HEPES, 0.2 EGTA, 8 NaCl, 2 MgATP, 0.3 NaGTP (pH 7.2–7.3), with an osmolarity of 270–280 mOsm.

Rheobase currents were measured under the current-clamp mode by injection of a series of 500-ms steps at 20-pA increments. The rheobase current was defined as the first current step capable of inducing one action potential^49^. For evoked firing of DMS neurons, action potentials were elicited by 500-ms current injections at 20-pA increments over a range of −50–+610 pA. The firing frequency of DMS neurons was calculated from the numbers of membrane voltage spikes generated over 500 ms from each current injection of +200–+610 pA. For measurement of the spontaneous firing of SNc neurons, cell-attached recordings were conducted in the voltage-clamp mode, as described previously^44^. Evoked action potentials were elicited by 500-ms stepped current injections at 30-pA increments from −150–+180 pA. The firing frequencies of SNc neurons were calculated from 0–+150 pA.

To measure AMPA-induced currents, AMPA (1 µM) was bath-applied for 30 s in the presence of picrotoxin (100 µM) to block inhibitory synaptic currents^15, 19^. AMPAR-mediated mEPSCs were measured in the present of 1 µM TTX, 100 µM picrotoxin, and 1.3 mM external Mg^2+^, as described previously^15, 19, 44^.

For recordings of GABAergic activity and transmission, the pipette solution contained (in mM): 125 CsCl, 6 NaCl, 10 HEPES, 1 EGTA, 0.6 Na_3_GTP, 2 MgATP, 10 QX-314.Cl, and 2 Na_2_CrPO_4_. Recordings were conducted at 32 °C. DNQX (20 µM) and CPP (10 µM) were added to the external solution to block AMPARs and NMDA receptors, respectively. To record GABA-induced currents, GABA (100 µM) was bath-applied for 30 s and holding currents were measured every 5 sec. sIPSCs were recorded for 3 min.

### Histology

Rats were perfused intracardially with 4% paraformaldehyde (PFA) in phosphate-buffered saline. The brains were post-fixed in 4% PFA overnight at 4 °C, followed by dehydration in 30% sucrose solution and cryostat frozen sectioning. The sections were post-stained with NeuroTrace green (1:100). A confocal laser-scanning microscope (A1si, Nikon) was used to image sections using a 594-nm laser for excitation of red RetroBeads and a 488-nm laser for NeuroTrace green.

### Statistical analysis

Electrophysiological data were analyzed using unpaired *t*-tests and two-way analysis of variance with repeated measurement (Two-Way RM ANOVA), followed by the Student-Newman-Keuls (SNK) *post hoc* test. All behavioral data were analyzed using one- or two-way RM ANOVA followed by the SNK test. mEPSCs and sIPSCs were analyzed using Mini analysis software (Synaptosoft Inc.). All data are expressed as the mean ± standard error of the mean.

## Electronic supplementary material

Supplementary information
