# Supplementary material for: Stroke triggers nigrostriatal plasticity and increases alcohol consumption in rats
Source: Sci Rep. 2017 May 31;7:2501. doi: 10.1038/s41598-017-02714-z (PMC5451385; doi:10.1038/s41598-017-02714-z)
Supplement: Supplementary file 1 — Supplementary information [file 41598_2017_2714_MOESM1_ESM.pdf]

Supplementary Information

Stroke triggers nigrostriatal plasticity and increases alcohol consumption in rats

Cathy C.Y. Huang[#], Tengfei Ma[#], Emily A. Roltsch Hellard[#], Xuehua Wang, Amutha Selvamani, Jiayi Lu, Farida Sohrabji, and Jun Wang*

Department of Neuroscience and Experimental Therapeutics, College of Medicine, Texas A&M University Health Science Center, Bryan, TX 77807, USA

[#]These authors contributed equally to this work.

* Corresponding author, jwang@medicine.tamhsc.edu

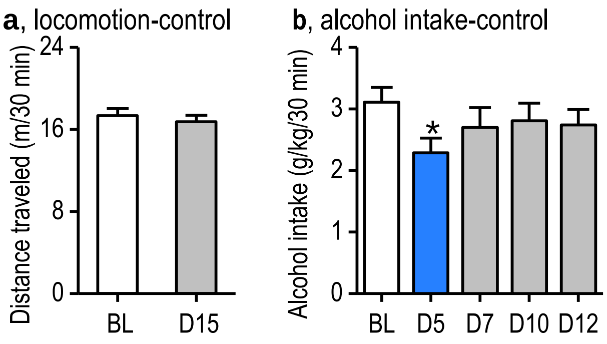

Supplementary Figure S1. Retrograde beads were infused into the dorsomedial striatum or substantia nigra pars reticulata of rats. Locomotor activity and alcohol intake were measured before (BL) and on indicated days after the infusion. (**a**) Stereotaxic infusion of retrograde beads did not alter locomotor activity on D15. Paired *t* test. n = 15 rats. (**b**) Stereotaxic bead infusion reduced alcohol intake on D5 but not on D7 and onwards. *$p < 0.05$ by One-Way RM ANOVA followed by SNK test. n = 23 rats.

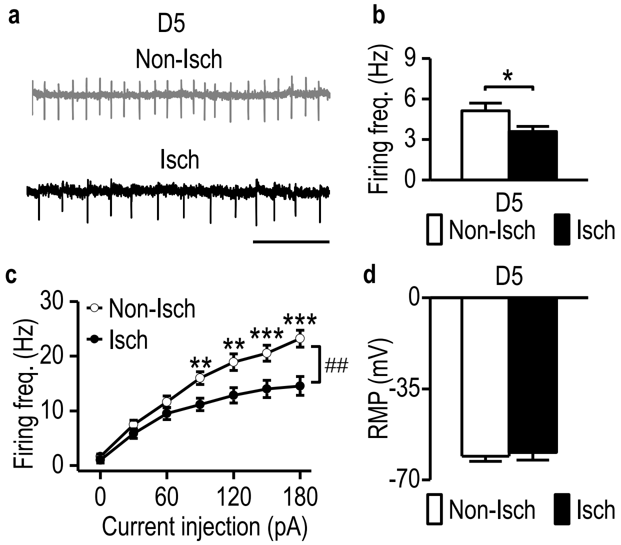

Supplementary Figure S2. Stroke decreases spike firing frequency of bead-negative SNc neurons in alcohol-drinking rats. Bead was infused into the DMS and stroke was induced the same as Figure 6. Bead-negative SNc neurons was recorded on D5 post-stroke. (**a**) Sample traces of spontaneous firing of bead-negative SNc neurons on the contralesional non-ischemic (Non-Isch) and ipsilesional ischemic (Isch) sides on D5. Scale bar: 1 s. (**b**) Bar graphs showing a decreased firing frequency in bead-negative SNc neurons on D5. $^{*}p < 0.05$ by *t* test. n = 9 neurons from 3 rats (Non-Isch) and 9 neurons from 3 rats (Isch). (**c**) Stroke decreased the excitability of bead-negative SNc neurons on D5. $^{\#\#}p < 0.01$ by Two-Way RM ANOVA; $^{**}p < 0.01$, and $^{***}p < 0.001$ vs. Non-Isch at the same intensity of current injection, post-hoc SNK test. n = 11 neurons from 3 rats (Non-Isch) and 12 neurons from 3 rats (Isch). (**d**) Bar graphs showing samilar resting membrane potentials (RMP) on the Non-Isch and Isch sides. n = 11 neurons from 3 rats (Non-Isch) and 12 neurons from 3 rats (Isch).

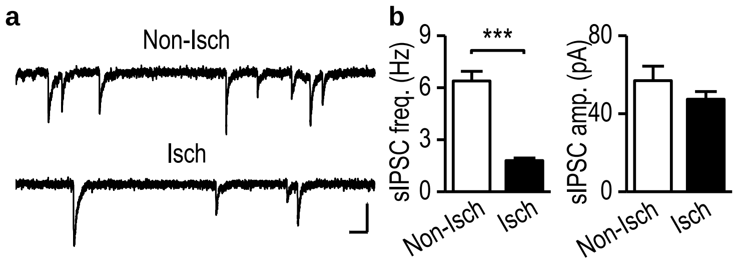

Supplementary Figure S3. Stroke-mediated DLS infarction reduces GABAergic inputs to DMS-projecting SNc neurons on D5 post-stroke. (**a**) Representative sIPSC traces in DMS-projecting SNc neurons on the Non-Isch and Isch sides on D5. Scale bars: 50 ms, 50 pA. (**b**) Stroke-induced DLS infarction reduced the frequency, but not the amplitude of sIPSCs in DMS-projecting SNc neurons. *Left*, bar graphs summarizing the average sIPSC frequencies. ***$p < 0.001$ by t test. Right, bar graphs displaying the average amplitudes. $p > 0.05$ by t test. n = 12 neurons from 5 rats (Non-Isch) and 14 neurons from 5 rats (Isch).
